# Image quality is resilient against tube voltage variations in post-mortem skeletal radiography with a digital flat-panel detector

**DOI:** 10.1038/s41598-021-87294-9

**Published:** 2021-04-08

**Authors:** S. Notohamiprodjo, K. M. Roeper, K. M. Treitl, B. Hoberg, F. Wanninger, L. Verstreepen, F. G. Mueck, D. Maxien, F. Fischer, O. Peschel, S. Wirth

**Affiliations:** 1 Department of Nuclear Medicine, Klinikum Rechts Der Isar, Technical University of Munich, Ismaninger Str. 22, 81675 Munich, Germany; 2 Department of Ophtalmology, Klinikum Rechts Der Isar, Technical University of Munich, Munich, Germany; 3 Department of Radiology, University Hospital of Munich, LMU Munich, Munich, Germany; 4 Agfa HealthCare Germany GmbH, Bonn, Germany; 5 Agfa-Gevaert HealthCare GmbH, Munich, Germany; 6 Agfa HealthCare N.V., Mortsel, Belgium; 7 Department of Radiology, HELIOS Klinikum München West, Munich, Germany; 8 Radiologie Zentrum München, Munich, Germany; 9 Institute of Forensic Medicine, LMU Munich, Munich, Germany; 10 Institute of Radiology, Schwarzwald-Baar Klinikum, Villingen-Schwenningen, Germany

**Keywords:** Radiography, Bone imaging

## Abstract

In recent phantom studies low-contrast detectability was shown to be independent from variations in tube voltage in digital radiography (DR) systems. To investigate the transferability to a clinical setting, the lower extremities of human cadavers were exposed at constant detector doses with different tube voltages in a certain range, as proposed in the phantom studies. Three radiologists independently graded different aspects of image quality (IQ) in a comparative analysis. The grades show no correlation between IQ and kV, which means that the readers were not able to recognize a significant IQ difference at different kV. Signal-to-noise and contrast-to-noise ratios showed no significant differences in IQ despite the kV-setting variations. These findings were observed from a limited kV range setting. Higher kV-settings resulted in lowest patient exposure at constant IQ. These results confirm the potential of DR-systems to contribute to standardization of examination protocols comparable to computed tomography. This may prevent the trend to overexpose. Further investigations in other body regions and other DR-systems are encouraged to determine transferability.

## Introduction

Digitization has taken its place in radiology, amongst others in skeletal radiography. Skeletal radiography still plays an essential role in the assessment of the skeletal status, metallic implants, injuries and other bony alterations, despite the superiority of Computed Tomography (CT). Along with dental and chest radiography, skeletal radiography is one of the most frequently performed x-ray examinations in the population of several countries^1–3^. The transition from screen-film to digital radiography (DR) led to the development and evolution of detector technology. Flat-panel detectors in direct digital radiography (DDR) acquire images by converting the incident x-ray energy into a digital signal almost instantaneously, by skipping the intermediary storage step. The mechanism of the energy transformation into a digital signal depends on the type of the detector. The detector used in DDR-systems acts as both the acquisition and conversion device. Flat-panel detectors are characterized with a higher dynamic range and quantum efficiency than screen-film^4^. To create an image of diagnostic quality by using flat-panel detector, a rigid control of exposure factors is no longer necessary, as was the case in film imaging. Furthermore, the wide dynamic range of such detector allows depiction of structures of varying attenuation in a single image. The acquired images can be digitally post-processed. To obtain optimal contrast and brightness, the use of a specific film-screen combination or set of radiographic technique parameters is therefore no longer necessary. This led to a decreased sensitivity for exposure variations on the one hand^5^. On the other hand the missing blackening of the film led to a trend to overexpose, the so-called “dose-creep”^6,7^. While the acquisition technology has advanced, it appears that guidelines have not optimized the radiographic technique factors for this new technology and continue to propose traditional technique parameters. This led to a large variety of examination protocols for all kind of body regions. Limited work has been conducted to optimize technique parameters to suit this new technology^8,9^. Advance of DDR-technology allows a significant image quality improvement by increasing the exposure factors. However, this is at the expense of increased radiation dose to the patient. According the “as low as reasonably achievable” (ALARA) principle, optimization rather than maximization of image quality in diagnostic radiography should be the main objective.

**Figure 1:**
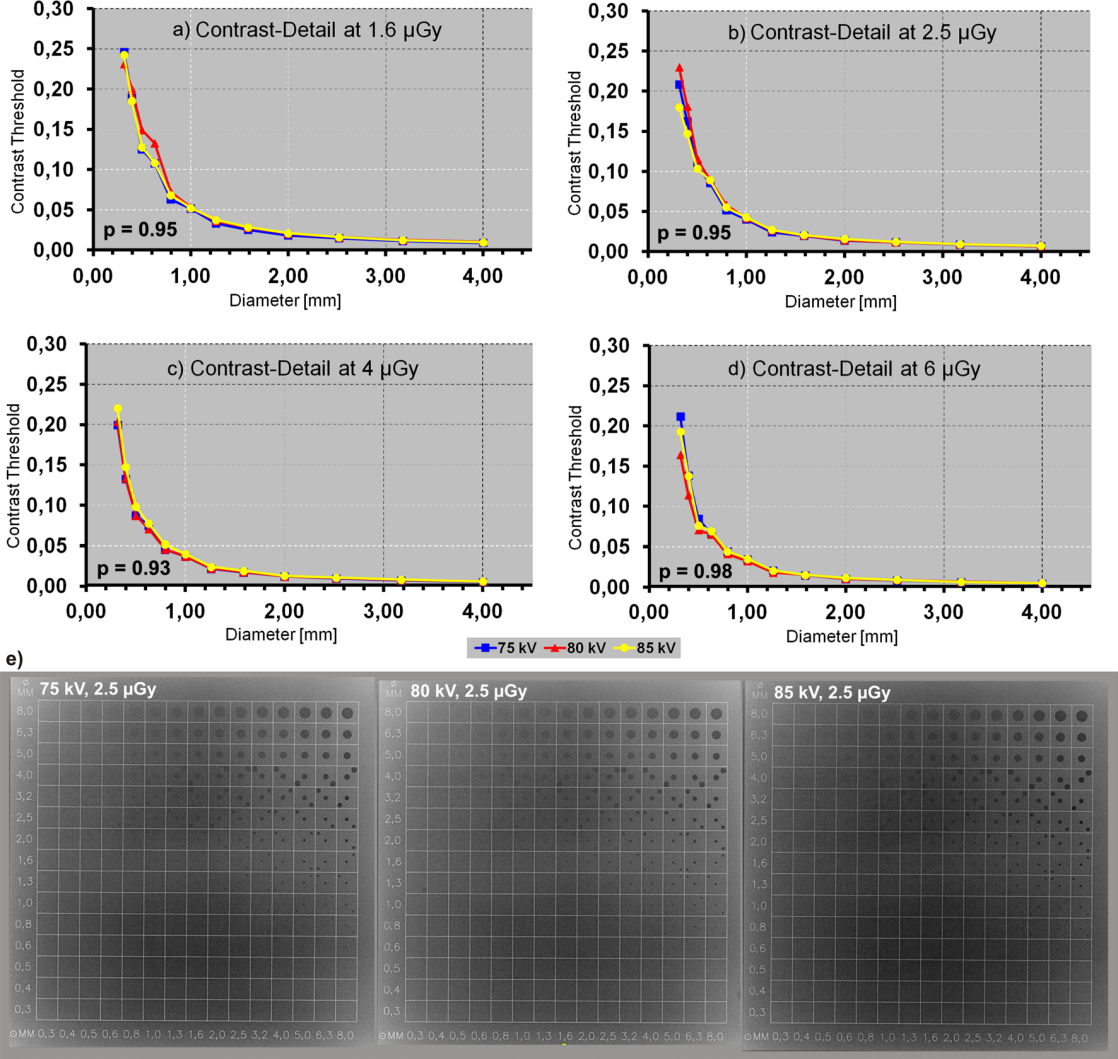
Phantom study: Low-contrast performance is not dependent on kV-setting in big proximal extremity examination simulation. Contrast-detail-curves (CDC) decrease with increasing low-contrast-structure (LCS) diameter. Threshold values for 5, 6.3 and 8 mm asymptotically approximate 0 (data hidden). (**a**) At an exposure dose of 1.6 µGy, no significant difference was found between the CDC of different kV-settings (p = 0.95). (**b**) At an exposure dose of 2.5 µGy, no significant difference was found between the CDC of different kV-settings (p = 0.95). (**c**) At an exposure dose of 4 µGy, no significant difference was found between the CDC of different kV-settings (p = 0.93). (**d**) At an exposure dose of 6 µGy, no significant difference was found between the CDC of different kV-settings (p = 0.98). (**e**) Example images of CDRAD 2.0 at 2.5 µGy at 75, 80 and 85 kV (from left to right). Reprinted from S. Notohamiprodjo et al. “Dependency of Low-Contrast Detail on Exposure Dose and Tube Voltage in Digital Flat-Panel Detector Radiography – a pre-clinical phantom study” Biomedical physics & engineering express 2018, 4(2)^10^ (c) IOP Publishing. Reproduced with permission. All rights reserved.

A recent phantom study calculating contrast-detail-curves for low-contrast-details in a DDR-system has shown an independence of the low-contrast detectability from tube voltage within a ± 5 kV range^10^ (Fig. 1). The tube voltage variation was applied on national guideline recommended examination protocols of small peripheral extremities (tube voltage 60 ± 5 kV) and large proximal extremities (tube voltage 80 ± 5 kV). The results indicate a potential for dose reduction while maintaining optimal image quality. Low contrast detail detectability is an overall parameter. However, the transition from phantom to human study i.e. from ideal condition to complex condition is difficult to link these results to performance in clinical use. In addition, non-linear image or post-processing is applied to clinical images, which is not applied to a contrast-detail phantom. At time of image acquisition parameters such as tube current, tube voltage, source-to-image distance can be adjusted and additional options of beam filtration and of scatter reduction can be chosen. Modification of each of these parameters directly affect patient dose and resultant image quality. To obtain high-quality diagnostic images, optimization of radiographic technique parameters is essential to be able to provide high-level patient care.

This study is to assess image quality in radiography of proximal and peripheral extremities in DDR and to examine the transferability of results from a previously published phantom study to clinical skeletal radiography. To avoid multiple exposures of patients, human cadavers referred for virtual autopsy were examined to simulate an application on living patients.

## Results

From a total of 70 corpses, 20 were finally included in the study population. The reasons of exclusion were affection of clinical routine (n = 23), infantile body (n = 18), advanced decay (n = 5), weight > 100 kg (n = 3) and destruction of legs (n = 1). 12 corpses were male, 8 female. The average age was 56.8 years, the average BMI 25.4. All corpses were fresh with an average elapsed time since death of approximately 1.8 days. Further population data are shown in Table 1.

**Table 1:** Study population.

Cadavers finally included	20 out of 70 cadavers, 12 male, 8 female
Reason of exclusion	affection of clinical routine (n = 23), infantile body (n = 18), advanced decay (n = 5), weight > 100 kg (n = 3), destruction of legs (n = 1)
Age	16 – 81 years, average 56.8 years
Height	159 – 186 cm, average 169.8 cm
Weight	51 – 98 kg, average 73.2 kg
Body Mass Index	15.1 – 34.9, average 25.4
Time elapsed since death	approximately 0.5 – 4 days, average 1.8 days
Location where the corpse was found	indoors (10), outdoors in the fresh air (9), unknown (1)
Circumstance of death	homicide/suicide (8), traffic accident (6), unclear (4), other accident (2)
Causes of death	craniocerebral injury (6), thorax/abdominal trauma (5), stab injury (4), disease (3), gunshot injury (3), strangulation (2), intoxication (1)

**Figure 2:**
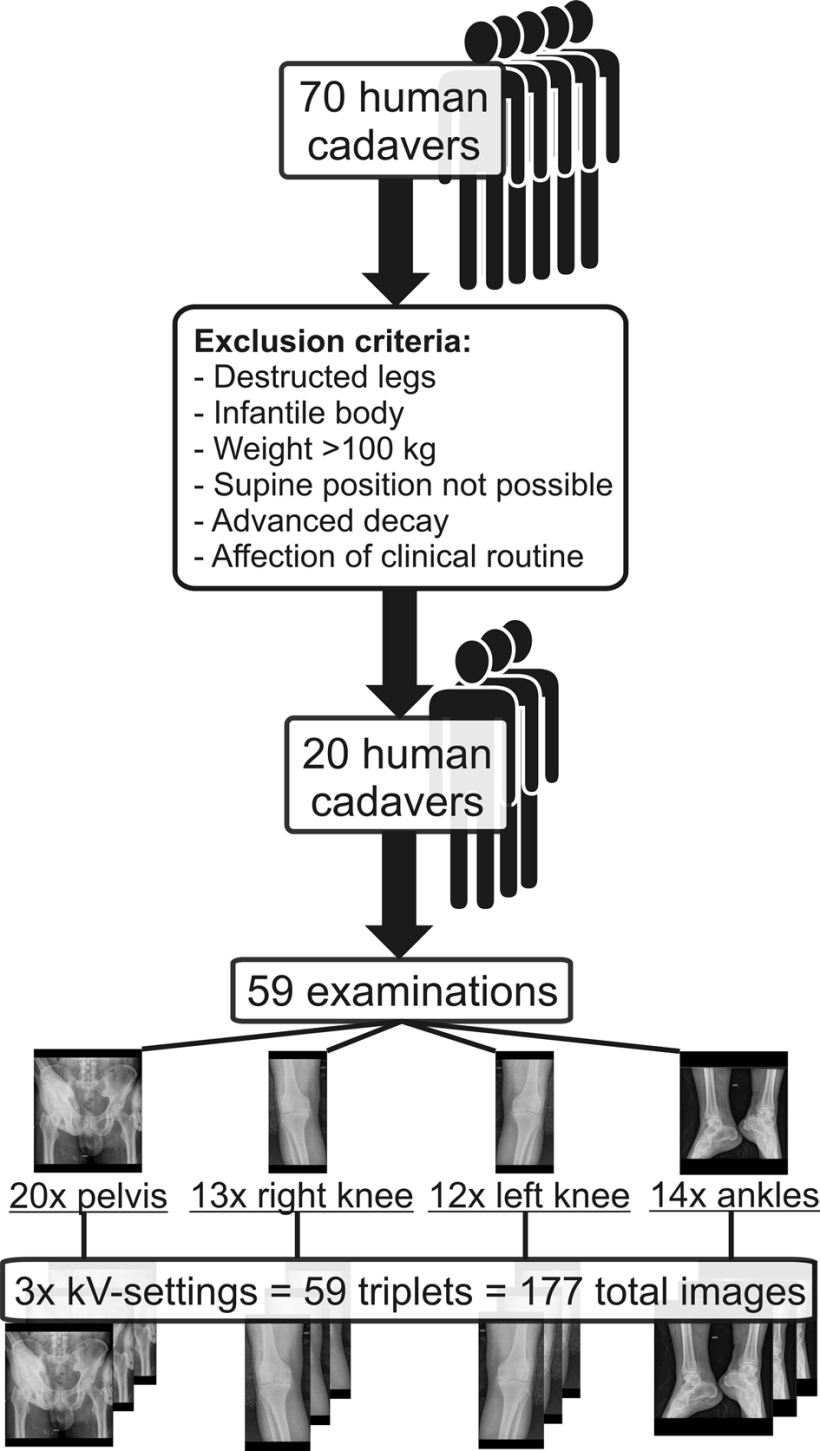
Study population selection process. From 70 human cadavers receiving post-mortem CT examination, a total of 20 human cadavers were referred to digital radiography. 59 examinations with a triplet of 3 kV-settings were performed including 20 pelvis, 13 right knees, 12 left knees and 14 ankles, resulting in a total number of 177 radiographs.

From 20 finally included corpses 20, 13, 12 and 14 triplets of pelvis, right knee, left knee and ankles were examined, resulting in 60, 39, 36 and 42 single images of pelvis, right knee, left knee and ankles (Fig. 2).

Dose-area-product (DAP) ranged from 38.4 to 500 cGy*cm^2^ in pelvis with the lowest average with a kV-setting of 85 (99.3 cGy*cm^2^). In knee DAP ranged from 0.86 to 22.9 cGy*cm^2^ with the lowest average with a kV-setting of 85 (9.5 cGy*cm^2^). In ankles DAP ranged from 0.58 to 5.28 cGy*cm^2^ with the lowest average with a kV-setting of 65 (2.7 cGy*cm^2^). Further average dose parameters are shown in Table 2. The deviation from the targeted exposure settings was −3.1% in average.

**Table 2:** Dose parameters.

kV	Pelvis			Knee			Ankles		
	75	80	85	75	80	85	55	60	65
Average DAP (cGy*cm^2^)	160.8	122.1	99.3	11.3	9.6	9.5	3.0	2.9	2.7
Effective dose (µSv)	466.4	354.2	288.1	1.13	0.96	0.95	0.30	0.29	0.27

### Image quality and distribution of grades

**Figure 3:**
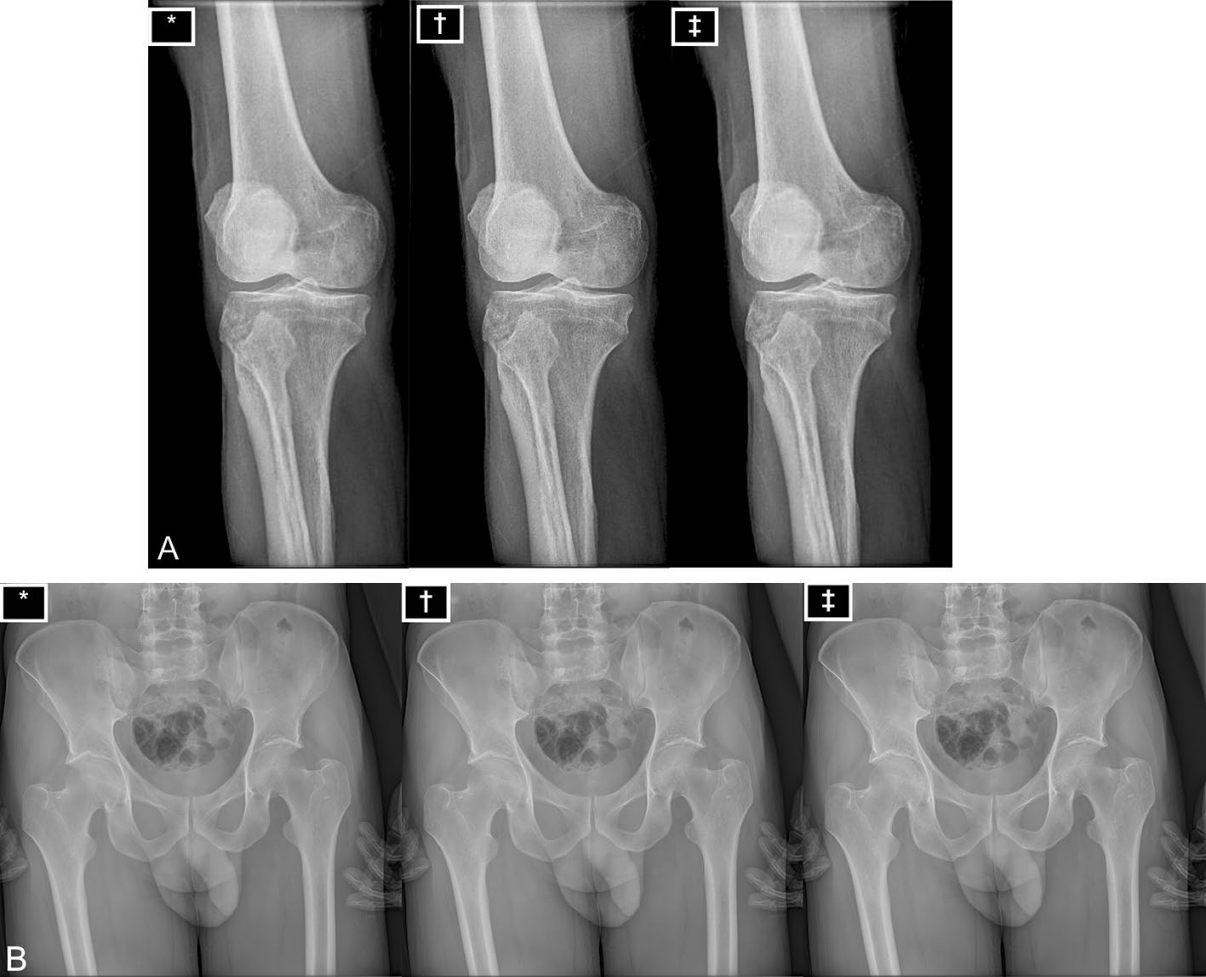
(**A**,**B**) Image triplet example. In this example, a right knee of a 43-year old male cadaver (**A**) and a pelvis of another 31-year old male cadaver (**B**). An image triplet of the identical examination with different kV-settings (± 5 kV) is displayed each on a particular monitor certified for diagnostic image reporting in a random order. Each image is marked by a symbol (*, †, ‡). Image * was performed with 85 kV, image † with 80 kV, image ‡ with 75 kV.

The visual impression of the images of the triplets with a variation of applied voltage of ± 5 kV was similar (Fig. 3). Fleiss Kappa analysis of the graded image triplets confirmed the arbitrary rating of the images. Agreement in the visual interpretation of the image quality within the radiologists was poor with κ = −0.049, p = 1.61 for hips, κ = −0.088, p = 1.88 for knees and κ = −0.059, p = 1.70 for ankles. The internal confidence of the grading of the images within the radiologists was high, with a Cronbach’s alpha coefficient of 0.81 for grading of hip, 0.70 for grading of knee and 0.72 for grading of ankles (Table 3). There was a high agreement between the observed probability (30.1% for hip, 27.5% for knee and 29.4% for ankle) and the expected probability (33.3%).

**Table 3:** Agreement in image quality.

Body part	κ-value	p-value	Cronbach $\alpha$	Friedman analysis	
				SNR	CNR
Hip	−0.049	1.61	0.81	p = 0.327	p = 0.654
Knee	−0.088	1.88	0.70	p = 0.071	p = 0.554
Ankle	−0.059	1.70	0.72	p = 0.113	p = 0.422

**Figure 4:**
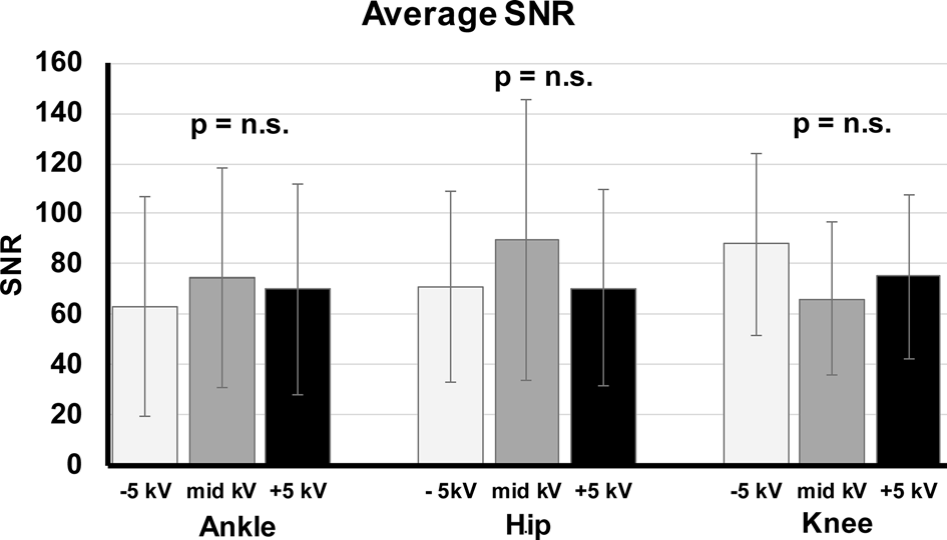
Average SNR of the image triplets of the ankles, hips and knees. SNR calculated in trabecular bone showed no significant changes within the image triplets (with ± 5 kV tube voltage variation) in ankle, hip and knee.

**Figure 5:**
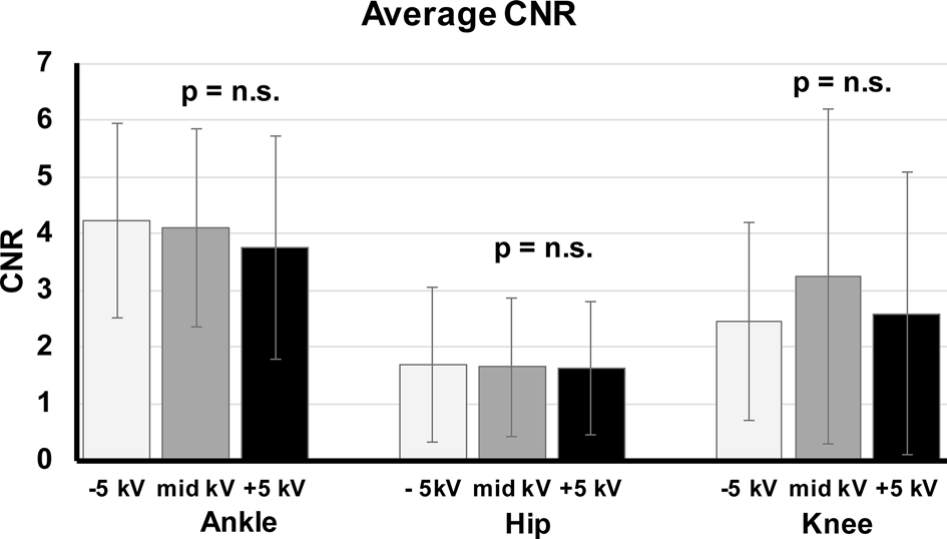
Average CNR of the image triplets of the ankles, hips and knees. CNR calculated between trabecular bone and soft tissue mantle showed no significant changes within the image triplets (with ± 5 kV tube voltage variation) in ankle, hip and knee.

The Friedman analysis (Table 3) of SNR within the image triplets showed no significant differences with p = 0.327 for hip, p = 0.071 for knee and p = 0.113 for ankle (Fig. 4). Similar results were obtained for CNR comparison within the image triplets with significant differences with p = 0.654 for hip, p = 0.554 for knee and p = 0.422 for ankle (Fig. 5).

**Figure 6:**
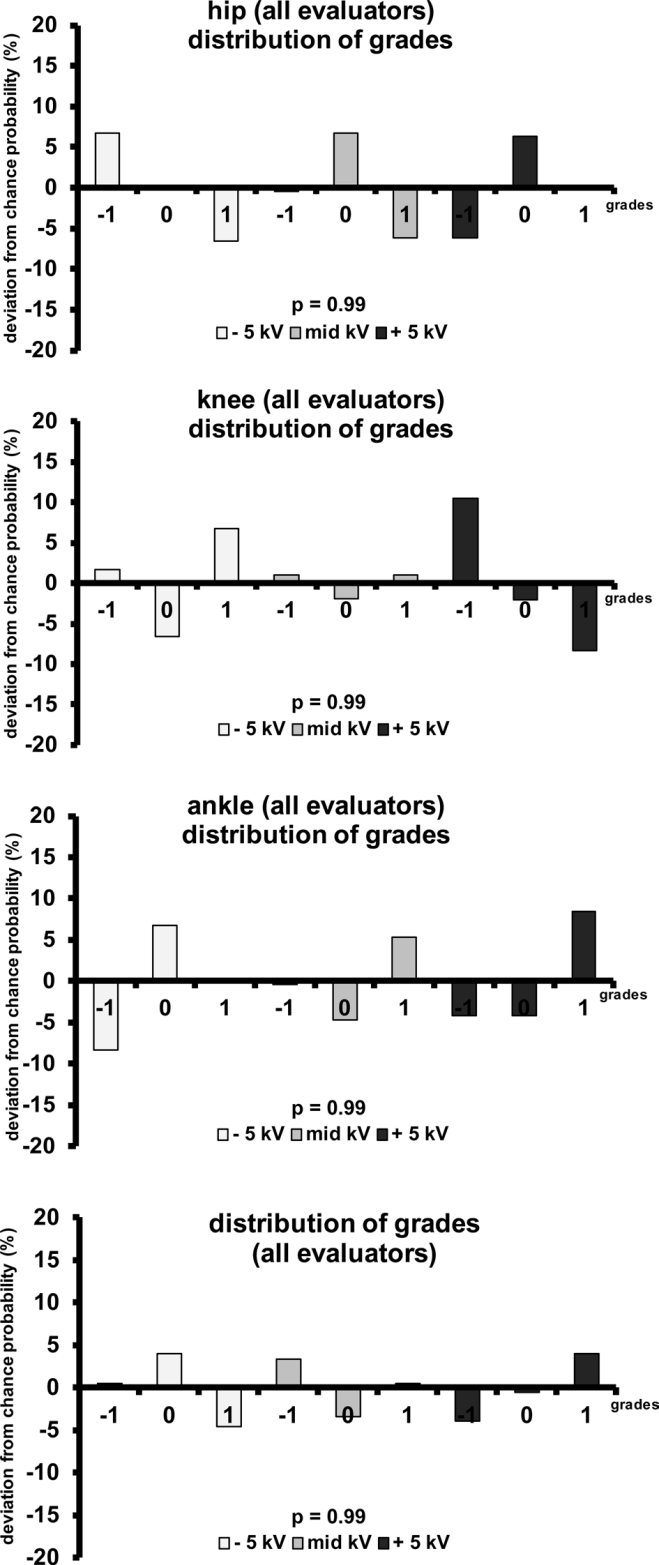
Distribution of grades given to each kV-setting itemized by body region. Three blinded evaluators graded the image quality (IQ) of a triplet with a 3-point scale (−1 = worst IQ of the triplet, 0 = middle IQ of the triplet, + 1 = best IQ of the triplet). The deviation from chance probability (33.3%) was insignificant in all body regions and all evaluators (p = 0.99). Agreement of the actual grades distribution with chance probability is highly probable (κ = −0.03), suggesting that evaluators were not able to differentiate kV-settings by IQ. The agreement between the evaluators was poor (κ = 0.01), indicating that the distribution of the grades given by the evaluators was random.

After pooling the distribution of grades given by the readers (Fig. 6), the deviations from chance probability for pelvis 75, 80 and 85 kV were 6.7%, −0.4% and −6.2% for worst IQ, 0.03%, 6.7% and 6.3% for middle IQ, −6.6%, −6.2% and 0.03% for best IQ. The deviations from chance probability for knee 75, 80 and 85 kV were 1.7%, 1.0% and 10.5% for worst IQ, −6.6%, −1.9% and −2.1% for middle IQ, 6.7%, 1.0% and −8.3% for best IQ. The deviations from chance probability for ankles 55, 60 and 65 kV were −8.3%, −0.4% and −4.1% for worst IQ, 6.7%, −4.7% and −4.1% for middle IQ, 0.03%, 5.3% and 8.4% for best IQ. P-values assessed for the difference between the actual grade distributions and chance probability itemized either by kV-setting or by grade order was always above 0.96.

## Discussion

This study’s aim was to assess the dependency of kV-settings and image quality of digital radiographs of big proximal and small distal joints. The results showed that the distribution of grades given to the overall IQ of each triplet image did not significantly deviate from chance probability. A Fleiss kappa near 0 showed a highly probable agreement between the actual grade distribution and chance probability. Usually it is expected that image quality decreases with increasing tube voltage, because of decreasing object contrast amongst others. However, the distribution of grades correlates rather with chance probability than with kV-setting. This indicates that the evaluators graded the image quality within the triplets in a random order, presumably because of no noticeable difference between the image qualities of the images within a triplet. Agreement between the readers was poor with Fleiss kappa near 0. This means that the grading of image quality made by the readers was independent from each other and based on chance probability rating. The absence of bias may exclude a systematic error. The quantitative assessment of IQ demonstrated no significant differences in SNR and CNR within the image triplets (with ± 5 kV tube voltage variation) in hip, knee and ankle. In conclusion image quality was not shown to be dependent of kV-setting (within a ± 5 kV range). This was observed in both settings for big proximal and small distal joints. These results confirmed the phantom data published previously^8^, indicating the successful transition from phantom study to preclinical post mortem study for peripheral digital skeletal radiography. Independency of image quality and kV-setting is important, because it allows a greater tolerance in the choice of technical examination parameters at constant image quality. This may contribute to a reduction of the variety of examination protocols in favour of general protocols for big or small sized body regions. This simplified selection of protocols may lead to an improved usability of DDR-systems with reproducible image quality despite of different patients or radiographers, to a more efficient clinical workflow and to the possibility to implement standardized examination protocols as known as in CT.

Recommendations for adjustments of technical examination parameters are discussed controversial in several studies investigating the performance of flat-panel detectors. On one hand a decrease of the tube voltage is recommended to yield an optimal image quality by utilizing the higher dose efficiency of flat-panel detectors^11,12^. On the other hand an increase of the tube voltage is recommended to reduce patient radiation exposure^13,14^, but at the expense of image contrast^15^. At present, the trend is to overexpose, which is a growing concern in digital radiography^6,7^. Other than in screen-film, overexposure leads to improved image quality in digital radiography while underexposure leads to increased image noise. This characteristic of digital radiography resulted in a risk to miss overexposure and led to a trend to increase patient exposure dose. Concordant to other reports^13,16^ average patient exposure doses were observed to be lowest at highest tube voltage in this study for both big proximal and small distal joints. In terms of the ALARA principle it is recommendable to choose the highest tube voltage of the kV-range evaluated in this study while maintaining sufficient image quality. An optimization of examination protocols with the lowest patient exposure dose and constant image resolution may contribute to the prevention of dose-creep.

In context to recent literature, this study’s results are interesting, because usually a decrease of tube voltage or increase of exposure dose is known to increase image quality.

A pre-clinical phantom study with a flat-panel detector showed that low-contrast performance was independent of kV-setting in a certain range^10^. This physical characteristic of the flat-panel detector may be a possible explanation for this study’s results that kV-setting had no apparent impact on image quality.

Another possible explanation for the independency of kV-setting in a certain range and exposure dose may be the impact of the post-processing on image quality. In particular, the post-processing used based on multiscale image contrast amplification (MUSICA) is known to reduce excess contrast and noise and to enhance subtle contrast and edges^5,17^. This study did not evaluate the extent of the impact on the raw data. A qualitative comparison of the raw images in this study was not applicable, because the settings were optimized for further post-processing and because the evaluators were not used to the visual impression of raw images. Furthermore other vendors developed a multi-frequency processing algorithm, such as Multi-Frequency-Processing (MFP) (Fuji) or Unified-Image-Quality (UNIQUE) (Philips) for example, enhancing image quality of radiographs in another extent than MUSICA^18^. However, post-processing by other vendors was not available for this study, being a limitation.

In this study an indirect-conversion DDR detector was used. Indirect-conversion and direct-conversion detectors are commonly used in DDR^5,19^. On the one hand indirect-conversion DDR detectors are usually built with a combination of a hydrogenated amorphous silicon (a-Si–H) layer and a thallium-activated caesium iodide (CsI-Tl) x-ray fluorescent layer. This combination contributes to an excellent x-ray absorption in the region of the spectrum most relevant to radiographic imaging and minimized image blur. On the other hand direct-conversion DDR detectors are built with an amorphous selenium (a-Se) x-ray photo-conductor layer. In comparison to CsI-Tl x-ray absorption efficiency is lower in a-Se and decreases with increasing beam energy more with a-Se than with CsI-Tl. However, a-Se absorber layers are independent of layer thickness and image sharpness, leading to improved spatial resolution^19^.

Apart from DDR, computed radiography is another technology used in radiographic imaging. Recent advances, such as needle-structured image plates (NIP), were reported to yield excellent image quality and detective quantum efficiency in comparison to conventional phosphor-storage image plates and flat-panel detectors^20,21^. However, computed radiography with image plates was not available for this study.

To further investigate if the results of this study derive from the characteristics of the detector used in this study or from image post-processing, a quantitative analysis of image quality and detective quantum efficiency with low-contrast and high-contrast detail phantoms and a comparison of these images with and without further post-processing are needed. Further studies with different types of flat-panel detectors and screen storage phosphor radiography systems are needed to evaluate the feasibility of general examination protocols.

In this study hip and knee were the sole representatives for big proximal joints, respectively ankle for small distal joints. The examination of the upper extremity was not reasonable because of the variable position in each body and limited possibility to reposition the extremity because of rigor mortis in some included bodies. To evaluate the feasibility of general examination protocols for big proximal and small distal joints, further body regions like shoulder, elbow, wrist or carpometacarpal joints need to be included to a follow-up study with the same experimental setting.

Besides the exact reasons of the independency of kV-setting of image quality need to be further explored, the huge amount and inconsistent examination protocols become obsolete thanks to the technological advances of digital radiography. They may pave the way to a simplified selection and the standardization of examination protocols.

The transition from screen-film to digital radiography led to a big variety of examination protocols. The current approach to adjust the examination parameters to the characteristics of digital radiography led to a trend to overexpose^6,7^. A simplified selection and the optimization of examination protocols is needed to prevent dose-creep and varying image quality depending on the performing radiographer and patient size. This comparative study’s aim to estimate the potential of flat-panel DDR-systems in combination with digital post-processing for general examination protocols in skeletal radiology revealed that image quality was independent from the used examination protocol with varying kV-settings. The results indicate the potential to reduce the selection of examination protocols to general protocols for big, respectively small joints with preference of lowest patient exposure at high kV-settings while maintaining sufficient image quality. General examination protocols may contribute to standardization as known as in CT. This is important to improve the clinical workflow, to reduce exposure dose and to increase diagnostic confidence thanks to a decrease of variability of image quality and visual impression of radiographs. Further studies to determine the transferability of general examination protocols on other digital radiography systems and other body regions are encouraged.

## Materials and methods

### Study population and special features of post-mortem examinations

This study was approved by the institutional ethical review board (Ethikkommission bei der Medizinischen Fakultät der LMU München, chairmen: Prof. Dr. W. Eisenmenger, Prof. Dr. R. M. Huber, Prof. Dr. C. Wendtner). All methods were performed in accordance with the relevant guidelines and regulations by the local board of medical council for quality control in diagnostic radiology and by the national federal office for radiation protection. Informed written consent of next of kin of deceased patients was waived by the institutional ethical review board. Virtual and conventional autopsy were ordered by the public prosecutor’s office. All human cadavers referred by the forensic medicine department for a post-mortem CT examination from August 2018 till September 2018 were initially included to the study population (Table 1).

To facilitate a smooth workflow, following exclusion criteria were implemented to the final study population receiving radiographs additional to CT: destruction of the legs, infantile bodies, weight more than 100 kg, supine position not possible, advanced decay, affection of the clinical routine. All data of the deceased such as age, weight, height, elapsed time since death, location of death, circumstances and cause of death were gathered from the dissection protocols provided by the forensic medicine department.

A flow chart of the acquisition of the study population is shown in Fig. 2.

### Acquisition of radiographs

**Figure 7:**
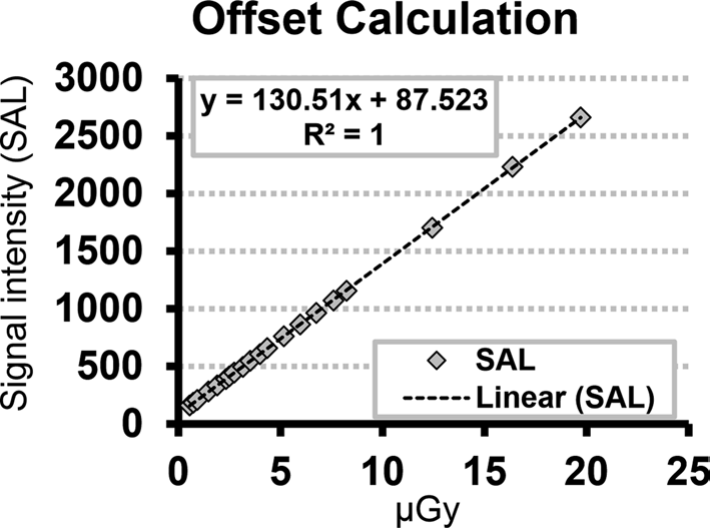
Detector response Pixel Value vs Entrance Air Kerma.

All conventional radiographs were performed with DX-D600 (AGFA HealthCare, Mortsel, Belgium), a direct digital radiography (DDR) system with fixed flat-panel detector Varian CsI (pixel size 139 µm) (Agfa HealthCare, Mortsel, Belgium). The curve of the detector response pixel value vs entrance air kerma is shown in Fig. 7.

The cadavers were examined in supine position. For orientation the CT survey images were inspected to assess the actual posture. A neutral positioning according the neutral zero method was applied as well as possible. The zipper of the body bag was shifted to an appropriate spot to avoid superposition with the bones in the radiographs. For collimation anatomical landmarks were palpated: iliac crest and greater trochanter for pelvis, patella for knee and mortise and heel for ankle.

The examination parameters for pelvis, single knee and both ankles complying with national regulations in radiation protection^22^ are subsumed in Table 4. All body regions were examined thrice with the same setting but different tube voltages as shown in Table 4. All images were post-processed with Multiscale image contrast amplification (MUSICA) 2 (Agfa HealthCare, Mortsel, Belgium) with default settings (Contrast = 0, Brightness = 0, Sharpness = 0). The total number of image triplets and single radiographs are displayed in Fig. 2.

**Table 4:** Examination protocols.

	Pelvis (a.p.)	Knee (a.p.)	Ankles (a.p.)
Tube voltage* ^27^	75 kV (28.8 mAs), 80 kV (19.0 mAs), 85 kV (13.5 mAs)	75 kV (7.52 mAs), 80 kV (5.60 mAs), 85 kV (4.48 mAs)	55 kV (2 mAs), 60 kV (1.6 mAs), 65 kV (1.25 mAs)
Exposure mode	Automatic exposure control	Automatic exposure control	Manual exposure
Anti-scatter grid	52 lines/cm, ratio 8:1, aluminium, f_0_ = 100 cm	52 lines/cm, ratio 8:1, aluminium, f_0_ = 100 cm	none
Source to image distance (SID)	1.15 m	1.15 m	1.15 m
Ionization chamber location	Left and right	middle	none

*Tube voltage on the DX-D 600 system was measured with a calibrated non-invasive multimeter prior to the measurements for 75 kV nominal and an accuracy of 3.1%. This is within the typical range of this type of DDR system and within the assumptions for the used kV-ranges.

### Documentation of radiation dose

Dose parameters were recorded in dose-area-product (DAP, [cGy*cm^2^]). Effective dose [µSv] was calculated from DAP multiplied with corresponding conversion factors (pelvis: 2.9 µSv/cGy*cm^2^, knee: 0.1 µSv/cGy*cm^2^; ankle: 0.1 µSv/cGy*cm^2^) as proposed by Hart and Wall^23^.

### Evaluation of image quality

A triplet consists of three images of the same body region of the same patient with different kV-settings. The images were displayed each on a particular monitor calibrated to the DICOM greyscale standard and officially certified for diagnostic image reporting (EIZO, Hakusan, Japan) in a random order (Fig. 3). The reading radiologists were not allowed to manipulate the DICOM greyscale window settings which were set identical for each monitor. To allow blinded evaluation, all patient data were anonymized and examination parameters were encoded according to a pattern unknown to the readers and the investigator.

Three radiologists with more than four years of experience in skeletal radiography independently assigned three grades to corresponding images of each kV-triplet according to overall image quality (IQ) (−1 = worst IQ of the triplet, 0 = middle IQ of the triplet, + 1 = best IQ of the triplet). Each grade can be given only once. For example, if one of the three images were graded with + 1, only the grades 0 and −1 are to be assigned to the two remaining images of the triplet. If one of the two remaining images of the triplet is then graded with −1, the last remaining image is automatically graded with 0. The radiologists were instructed to stick to the criteria based on the guidelines published by the American College of Radiology ^24^ and European Commission ^25^ rather than to subjective criteria such as eye pleasing appearance of the image. In this study the comparison of IQ within the triplets and ranking of IQ in order of quality within the triplet is essential rather than the grading of IQ of each image. Furthermore, a quantitative assessment of IQ in terms of Signal-to-Noise-Ratio (SNR) and Contrast-to-Noise-Ratio (CNR) derived from specific anatomical structures of the images was performed.

SNR was defined as:

$$SNR = \frac{{\mu_{target} }}{{\sigma_{bg} }}$$

CNR was defined as:

$$CNR = \frac{{\left| {\mu_{target} - \mu_{ref} } \right|}}{{\sqrt {\sigma_{target}^{2} + \sigma_{ref}^{2} } }}$$

**Figure 8:**
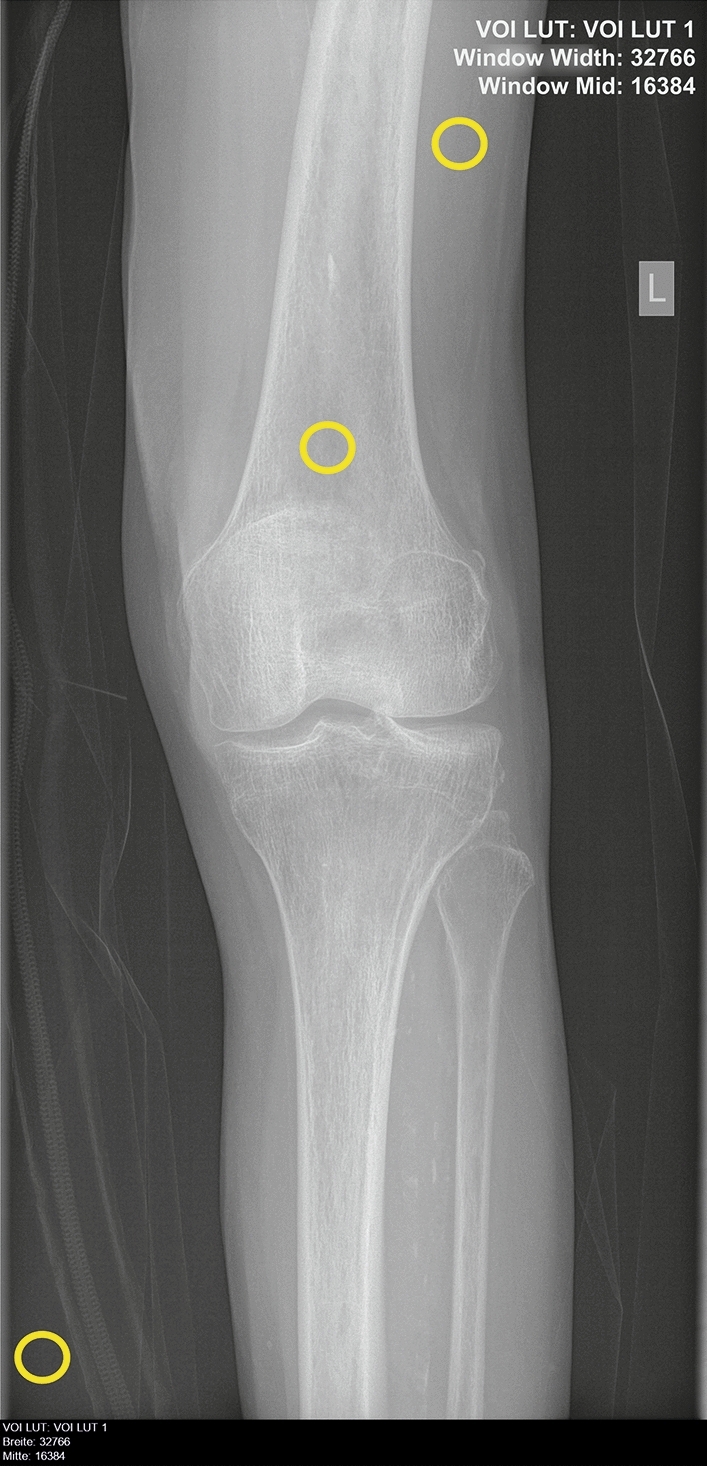
Location of ROIs for quantitative analysis. Target ROI = Trabecular bone, Reference ROI = Soft tissue, Background ROI = Background region.

µ_target_ and µ_ref_ denote the mean of signal in a certain region of interest (ROI) at the target structure and the reference (ref) tissue, respectively. $\sigma$_target,_ $\sigma$_ref_ and $\sigma$_bg_ represent the standard deviation of the according signal in the ROI of the target structure and reference tissue and background region (bg), respectively. The ROI were manually chosen areas of 64 × 64 pixels. For the background region the ROI was placed outside the object. Target structure was primarily trabecular bone, reference tissue was the soft tissue mantle. No ROI were placed in regions with superposition with other materials (Fig. 8).

### Statistical evaluation

All statistical analyses were carried out with “IBM SPSS Statistics 23” (IBM Corporation, Endicott, NY, USA). Significance level $\alpha$ was defined at 0.05 with p < $\alpha$.

We assume that radiologists perceive no difference in image quality in case of a true null hypothesis “variations in tube voltage have no significant impact on the detectability of low- and high-contrast structures”. If no difference in image quality is perceptible, radiologists are assumed to assign the grades randomly, leading to a distribution of grades according the chance probability. The estimated distribution by chance of the grade for each image would be 33.3%.

If kV-setting has a significant impact on the detectability of low- and high-contrast structures, the radiologists are assumed to perceive a varying image quality within a triplet, for example worse contrast and thus worse delineation of small details such as trabecular bone or streaks of fat within the soft tissue in higher kV-settings. In this case the distribution of grades would significantly differ from chance probability and evaluators would show a good agreement in their distribution of grades.

The deviations of the actual distribution of the given grades from the chance probability are displayed as bar charts. Significance of the difference between the actual grade distributions from chance probability was assessed with t-test. A p-value of < 0.05 was considered to be statistically significant. Thus, insignificance may hint to a grade distribution by chance.

To assess either the agreement between the readers or the agreement between the actual grade distribution and the chance probability to exclude a systematic error, Fleiss Kappa (κ) was calculated. The interpretation of κ values was defined by Landis & Koch^26^. Internal consistency of the grading was assessed using Cronbach’s alpha coefficient. A low value for Cronbach’s alpha hints to an inconsistency in the evaluator’s judgement, e.g. because of external influences or negligence. A high value for Cronbach’s alpha confirms the consistency in the evaluator’s judgement. Comparison of the IQ grading of the triplet between the readers was performed with Kruskall-Wallis-Test. SNR and CNR of the triplet were compared via Friedmann-Test.

## References

[1] Regulla DF, Eder H (2005). Patient exposure in medical X-ray imaging in Europe. Radiat. Prot. Dosimetry..

[2] Le Coultre R (2016). Exposure of the Swiss Population by Radiodiagnostics: 2013 Review. Radiat. Prot. Dosimetry..

[3] Zenone F (2012). Effective dose delivered by conventional radiology to Aosta Valley population between 2002 and 2009. Br J Radiol.

[4] Yaffe MJ, Rowlands JA (1997). X-ray detectors for digital radiography. Phys. Med. Biol..

[5] Körner M (2007). Advances In Digital Radiography: Physical Principles and System Overview. Radiographics.

[6] Mothiram U, Brennan PC, Lewis SJ, Moran B, Robinson J (2014). Digital radiography exposure indices: a review. J. Med. Radiat. Sci..

[7] Ching W, Robinson J, McEntee M (2014). Patient-based radiographic exposure factor selection: a systematic review. J. Med. Radiat. Sci..

[8] Steffensen C, Trypis G, Mander GTW, Munn Z (2019). Effectiveness of adjusting radiographic technique parameters on image quality in direct digital radiography – a systematic review protocol. JBI Database System Rev. Implement. Rep..

[9] Phillips KL (2015). Radiographic skeletal survey for non-accidental injury: systematic review and development of a national New Zealand protocol. J. Med. Imaging Radiat. Oncol..

[10] Notohamiprodjo S (2018). Dependency of Low-Contrast Detail on Exposure Dose and Tube Voltage in Digital Flat-Panel Detector Radiography – a pre-clinical phantom study. Biomed. Phys. Eng. Express.

[11] Uffmann M, Schaefer-Prokop CM (2009). Digital Radiography: The Balance between image quality and required radiation dose. Eur. J. Radiol..

[12] Tingberg A, Sjostrom D (2005). Optimisation of image plate radiography with respect to tube voltage. Radiat. Prot. Dosimetry..

[13] Doherty P, O'Leary D, Brennan PC (2003). Do CEC guidelines under-utilise the full potential of increasing kVp as a dose-reducing tool?. Eur. Radiol..

[14] Hamer OW, Völk M, Zorger N, S., F. & M., S. (2003). Amorphous silicon, flat-panel, x-ray detector versus storage phosphor-based computed radiography: contrast-detail phantom study at different tube voltages and detector entrance doses. Investig. Radiol..

[15] Geijer H, Persliden J (2005). Varied tube potential with constant effective dose at lumbar spine radiography using a flat-panel digital detector. Radiat. Prot. Dosimetry..

[16] Strotzer M (1998). Amorphous Silicon, Flat-Panel, X-Ray Detector versus Screen-Film Radiography: Effect of Dose Reduction on the Detectability of Cortical Bone Defects and Fractures. Invest. Radiol..

[17] Moore CS, Liney G, Beavis AW, Saunderson JR (2007). A method to optimize processing algorithm of computed radiography system for chest radiography. Br. J. Radiol..

[18] Schaefer-Prokop CM, De Boo DW, Uffmann M, Prokop M (2009). DR and CR: Recent advances in technology. Eur. J. Radiol..

[19] Cowen AR, Kengyelics SM, Davies AG (2008). Solid-state, flat-panel, digital radiography detectors and their physical imaging characteristics. Clin. Radiol..

[20] Wirth S, Treitl M, Reiser MF, Körner M (2008). Imaging Performance with Dfferent Doses in Skeletal Radiography: Comparison of a Needle-structured and a Conventional Storage Phosphor System with a Flat-Panel Detector. Radiology.

[21] Körner M (2006). Depiction of Low-Contrast Detail in Digital Radiography - Comparison of Powder- and Needle-Structured Storage Phosphor Systems. Invest. Radiol..

[22] Bundesärztekammer. Leitlinie der Bundesärztekammer zur Qualitätssicherung in der Röntgendiagnostik - Qualitätskriterien röntgendiagnostischer Untersuchungen. (2007).

[23] Hart, D. & Wall, B. F. Radiation Exposure of the UK Population from Medical and Dental X-ray Examinations. *National Radiological Protection Board* **NRPB-W4** (2002).

[24] American College of Radiology, A. ACR-SPR-STR Practice Parameter for the Performance of Chest Radiography. *Available at: *https://www.acr.org/-/media/ACR/Files/Practice-Parameters/ChestRad.pdf (2017).

[25] European Commission. European Guidelines on Quality Criteria for Diagnostic Radiographic Images. *EUR 16260 EN* (1996).

[26] Landis JR, Koch GG (1977). The measurement of observer agreement for categorical data. Biometrics.

[27] Bath M, Svalkvist A, von Wrangel A, Rismyhr-Olsson H, Cederblad A (2010). Effective dose to patients from chest examinations with tomosynthesis. Radiat. Prot. Dosimetry..

